# Survey on the Presence of Bacterial, Fungal and Helminthic Agents in Off-Leash Dog Parks Located in Urban Areas in Central-Italy

**DOI:** 10.3390/ani11061685

**Published:** 2021-06-05

**Authors:** Valentina Virginia Ebani, Simona Nardoni, Stefania Ciapetti, Lisa Guardone, Enrico Loretti, Francesca Mancianti

**Affiliations:** 1 Department of Veterinary Sciences, University of Pisa, Viale delle Piagge 2, 56124 Pisa, Italy; simona.nardoni@unipi.it (S.N.); ciapettivet@gmail.com (S.C.); lisa.guardone@for.unipi.it (L.G.); francesca.mancianti@unipi.it (F.M.); 2 Centre for Climate Change Impact, University of Pisa, Via del Borghetto 80, 56124 Pisa, Italy; 3 UFC Igiene Urbana, USL Toscana Centro, Viale Corsica 4, 50127 Firenze, Italy; enrico.loretti@uslcentro.toscana.it; 4 Interdepartmental Research Center “Nutraceuticals and Food for Health”, University of Pisa, via del Borghetto 80, 56124 Pisa, Italy

**Keywords:** dogs, off-leash parks, feces, bacteria, fungi, parasites

## Abstract

**Simple Summary:**

Off-leash dog parks are designated, generally fenced, public spaces where dogs can move freely under the supervision of their owners. These areas, allowing animals to socialize and run free, play a fundamental role in dogs’ welfare. However, such environments may be a source of different pathogens, even zoonotic, excreted by the attending animals. The present study evaluated the occurrence of bacterial, fungal, and parasitic pathogens in off-leash dog parks located in Florence (central Italy). *Yersinia* spp., *Listeria innocua, Toxocara canis* eggs and *Ancylostoma caninum/Uncinaria stenocephala* eggs were found in canine feces. Keratinophilic geophilic fungi (mostly *Microsporum gypseum/A. incurvatum, Microsporum canis* in a single case) were recovered from soil. *Trichosporon* sp. and *Geotrichum candidum* were isolated from two water samples. The obtained results suggest that, despite the not negligible canine fecal contamination of selected areas (feces were found in 88.5% of the parks), attending dogs did not act as important carriers for the investigated pathogens, although examined off-leash dog parks may represent a risk for the spreading of some dermatophytoses to both pets and their owners. Thus, in a One-Health perspective, periodical examinations to detect the main bacteriological, parasitological and mycological pathogens in different samples collected in off-leash dog parks are recommended.

**Abstract:**

Off-leash dog parks are designated public spaces where dogs can move freely, under their owners’ supervision. These areas, allowing animals to socialize and move freely, are fundamental for dogs’ welfare. However, different pathogens, even zoonotic, may be excreted by the attending animals and contaminate the environment. The aim of the present study was to verify the occurrence of bacterial, fungal and parasitic pathogens in off-leash dog parks located in Florence (central Italy). Between March and May 2019, 83 fecal samples, 43 soil samples and 23 water samples (from fountains and puddles) collected from 26 off-leash fenced areas were examined. Fecal samples scored positive for *Yersinia* spp. (*n* = 7), *Listeria innocua* (*n* = 4), *Toxocara* *canis* eggs (*n* = 2) and *Ancylostoma caninum/Uncinaria stenocephala* eggs (*n* = 1). Keratinophilic geophilic fungi (mostly *Microsporum gypseum /A. incurvatum*) were recovered from 43 soil samples belonging to 23 out of 26 parks, along with *Microsporum canis* in a single case. *Prototheca* spp. was never isolated from water samples, while *Trichosporon* sp. was cultured in two cases, alone and in association with *Geotrichum candidum*. These results show that dogs did not act as important carriers for the investigated bacterial and parasitic pathogens, although examined areas may represent a risk for the spreading of some dermatophytoses to both pets and their owners. Periodical examinations to assess the main bacteriological, parasitological and mycological pathogens in different samples collected in off-leash dog parks should be carried out in a One-Health perspective.

## 1. Introduction

Off-leash dog parks are designated public spaces where dogs, without a leash, can move freely under their owners’ supervision [1]. Off-leash dog parks are of particular interest in health and welfare promotion because they allow physical activity and social networking [1], even though some dogs may show some stress-related behaviors [2].

Besides potential psyco-physical consequences for dogs, the frequentation of these public areas may raise some concerns about public safety and nuisance [3], as dogs carrying pathogens may excrete them and become a source of infections for other animals.

In fact, dogs can be infected, even asymptomatically, by several zoonotic enteric bacteria and excrete these agents in their feces. Among them, the most relevant and frequent belong to the genus *Salmonella*, *Campylobacter*, *Yersinia* and *Listeria*. Dogs can be infected by *Salmonella enterica*, a Gram-negative, rod-shaped, flagellated and facultative anaerobe bacterium. Salmonellosis is one of the most common zoonoses in Europe [4], and pet animals, other than farm animals, are involved in epidemiology.

*Campylobacter* sp. are Gram-negative rods with a polar flagellum; they have been recognized as a common cause of acute diarrhea in humans since 1977 [5] and were subsequently cultured from dog feces [5,6]. The genus *Campylobacter* currently includes over 30 different species and subspecies. *C. upsaliensis*, *C. jejuni* and *C. helveticus* are the most frequently involved in canine infections [7].

*Yersinia enterocolitica*, another important zoonotic agent, can cause infection in canine populations. This is a Gram-negative, bacillus-shaped bacterium; it has been divided into more than 70 serotypes based on differences in the structure of the somatic antigen and into six biotypes based on its biochemical characteristics [8].

Even though studies about *Listeria* sp. infection in canine populations are very scant, it has been proven that these agents can be present in dogs’ feces. The genus *Listeria* includes Gram-positive, no-spore forming, rod-shaped, facultative intracellular bacteria responsible for infections in several mammal species, including humans. Currently, 21 species are included in this genus [9], but *L. monocytogenes* is the main agent associated with illness in animals and humans [10].

Furthermore, dogs can also harbor several zoonotic parasites. Among them, geohelminths such as *Toxocara canis*, *Trichuris vulpis* (whipworm) and *Ancylostoma* caninum (hookworm) are frequently found to contaminate the environment, and canine fecal pollution can predispose to infection people attending park, as well as their pets. Traversa and coll. [11] excellently reviewed the occurrence of eggs of canine intestinal nematodes in urban areas, also pointing out the possibility that eggs of *T. canis* and *A. caninum* may represent a risk of infection for other animal species, acting as paratenic hosts for such parasites.

Moreover, animals shed keratinized material that becomes hair baits and are attacked by keratinophilic fungal species, which tend to proliferate when keratin is present. Keratinophilic fungi are specialized keratin-degrading organisms [12]. Some species within the group can infect and degrade keratinic tissue off-host only (strictly geophilic species), while others may degrade keratinic tissue also on the host (geophilic dermatophytes), being capable of attacking hairs, skin, nail and other keratinized tissues, provoking ringworm [13].

*Prototheca* spp. are achlorophyllic algae occurring in soil, water, sewage and vegetative matter. Within the genus, *P. bovis*, *P. ciferrii*, *P. wickerhamii*, and *P. zopfii* are the species involved in canine disease [14]. Canine protothecosis consists of severe colitis that frequently turns into a fatal systemic affection. Moreover, *P. wickerhamii* and *P. zopfii* are the most frequently involved species in human protothecosis [15,16,17], a life-threatening condition, especially in immunocompromised people. These affections, although accounted as rare, are found to be emergent diseases [14,16,18], and environmental infection is considered the main route for intestinal and systemic disease.

Finally, yeasts are proven important pathogens both in animals and humans, and most of them can occur as saprophytes in the environment, being able to grow on not viable organic matter. In a recent study, thermotolerant strains of *Candida* spp. (*C. guilliermondii*, *C. famata* and *C. parapsilosis*) with low sensitivity to some important antimycotic drugs used to treat human patients, were recovered from decayed tree parts in green urban areas [19]. These fungal species can induce life-threatening infections, mostly in immunocompromised people and are also reported as emergent pathogens in recent years [20].

The aim of the present investigation was to verify the presence of the main bacterial, helminthic and fungal canine agents in off-leash dog parks located in different urban areas of Florence city (Central Italy). The study was carried out from a One Health perspective; in fact, it aimed to evaluate whether the selected areas can be a source of infections for dogs and humans.

For this purpose, stool samples were collected to culture bacteria (*Campylobacter* spp., *Salmonella* spp., *Yersinia* spp. and *Listeria* spp.) and to search for helminthic diagnostic stages; soil samples were collected to evaluate the presence of keratinophilic fungi, and water samples were taken to detect *Prototheca* spp. and potentially pathogenic yeasts.

## 2. Material and Methods

### 2.1. Study Areas

The survey was carried out between March and May 2019. Twenty-six of the 43 off-leash dog parks (60.5%) located in Florence city were included in the study. Parks were randomly chosen with the aim to sample several dog areas equal to more than half of their total number and to cover more than half of the square meters of public areas destined for this purpose.

### 2.2. Sampling

In total, 43 soil samples, 83 fecal samples and 23 water samples were collected. All fecal samples present in the parks were collected. Soil and fecal samples were placed in clean plastic containers, whereas water samples were placed in sterile test tubes. Fecal specimens collected were fresh to allow correct processing, mostly to avoid the occurrence of false-negative parasitological results when strongylids would occur. Anyway, feces were removed by the city every 3 days, about. The samples found cannot be attributed to known animals, so the occurrence of the feces of the same dog cannot be excluded.

To specifically search for keratinophilic geophilic fungi, soil specimens (about 300 g) were collected from the superficial ground layer, with a depth not exceeding 3 cm, under the hedges, at the foot of the trees or under the benches, mainly choosing grass areas, possibly protected from direct sunlight, that should exert an antimycotic effect. Water specimens were sampled from water collections around the fountain (overflowed from the fountain basin), except for 4 parks where the fountain was not present and for 5 parks where puddles of rainwater were sampled. Puddles were evaluated to be present within about a week. All water specimens were drawn far from feces.

Each sample was recorded reporting date and the coded name of the off-leash park of collection. All samples were refrigerated at 4 °C and delivered within 24 h to the Department of the Veterinary Sciences of the University of Pisa.

**Figure 1:**
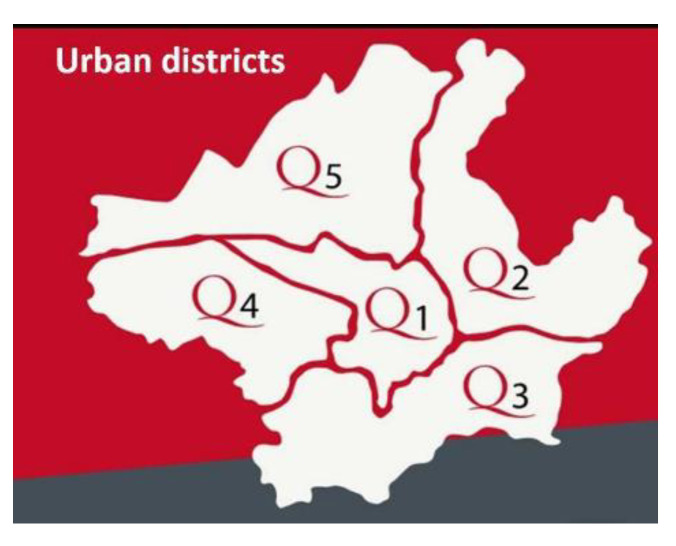
The distribution of the urban districts in Florence city.

**Figure 2:**
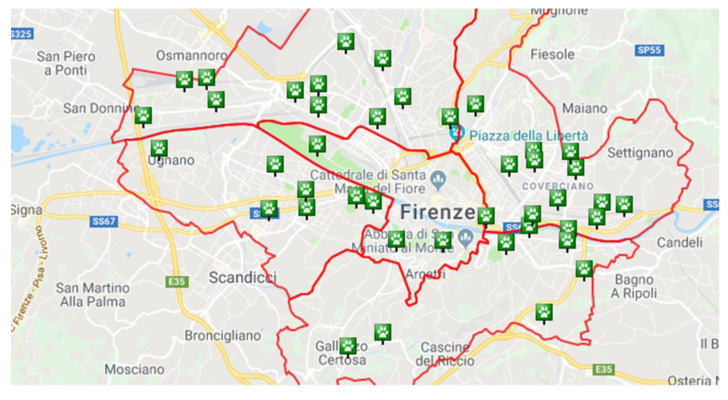
The distribution of the canine off-leash parks in the urban districts.

Table 1, Figure 1 and Figure 2 report data about the areas where samples were collected.

### 2.3. Bacteriological Examinations

#### 2.3.1. *Salmonella* spp.

*Salmonella* spp. isolation was executed from each fecal sample following the procedures previously described [21]. Briefly, about 3 gr of feces was incubated in 10 mL of buffered peptone water at 37 °C for 24 h. One ml of this culture was transferred into 10 mL of Selenite Cystine Broth (Oxoid Ltd., Basingstoke, UK) and 1 mL into 10 mL of Rappaport Vassiliadis Broth (Oxoid Ltd., Basingstoke, UK). The tubes were incubated at 37 °C for 24 h and at 42 °C for 24 h, respectively. One loopful from each broth culture was streaked onto Salmonella-Shigella Agar (Oxoid Ltd., Basingstoke, UK) and Brilliant Green Agar (Oxoid Ltd., Basingstoke, UK) plates. After incubation of the plates at 37 °C for 24 h, suspected colonies were submitted to biochemical characterization.

#### 2.3.2. *Campylobacter* spp.

The isolation of *Campylobacter* spp. from each fecal sample was carried out with an enrichment step in tubes containing Tryptone Soy Broth (Oxoid Ltd., Basingstoke, UK) liquid medium added with 5% horse blood and incubated at 37 °C for 4 h, then at 42 °C for 24 h.

A loop of each broth culture was streaked onto dishes containing Campylobacter Blood-Free selective agar base added with CAT selective supplement (Oxoid Ltd., Basingstoke, UK). Plates were incubated at 37 °C for 48 h in microaerophilia (5% oxygen, 10% CO_2_) [22].

#### 2.3.3. *Yersinia* spp.

After enrichment for each fecal sample in Peptone Sorbitol Bile Broth (Oxoid Ltd., Basingstoke, UK) for 21 days at 4 °C, a loop of the broth culture was sub-cultured onto Cefsulodin Irgasan Novobiocin (CIN) Agar, and the plates were incubated at 30 °C for 48 h. Suspected colonies were submitted to biochemical tests to determine the species [23], and confirmation was performed using the API20E biochemical gallery (bioMerieux, Marcy l’Etoile, France).

*Yersinia enterocolitica* isolates were successively characterized on the basis of biochemical tests to distinguish the biotype [24].

#### 2.3.4. *Listeria* spp.

*Listeria* strains were isolated according to the ISO 11290 method with modifications. The intestinal feces were introduced into 10 mL Half-Fraser broth (Oxoid Ltd., Basingstoke, UK) and incubated at 30 °C for 24 h. Subsequently, 0.5 mL of the primary enrichment cultures were transferred to 4.5 mL Fraser broth and incubated at 37 °C for 48 h. A loopful of secondary enrichment was streaked onto Chromogenic Listeria Agar (Oxoid Ltd., Basingstoke, UK) and incubated at 37 °C for 24–48 h. After incubation, the colonies suspected of being *Listeria* spp. based on color and morphology were selected and identified by API Listeria test (bioMérieux, Marcyl’Etoile, France) [25].

### 2.4. Parasitological Examinations

Fecal samples were examined for proglottids, nematodes and/or fragments of parasites, then microscopically screened by flotation test (2 g of feces) with a low-density solution (s.g. 1.2) to evaluate the occurrence of helminth eggs and/or protozoal cysts and oocysts [26].

### 2.5. Mycological Examination

About 30 g of each soil sample were put into Petri dishes (15 cm diameter), using a modification of Orr’s hair bait technique as reported by Caretta et al. [27]. Briefly, sterile feathers, and horse’s and child’s hair, cut by sterile scissors, were put on the soil moistened with a solution of cycloheximide (1 g/L). Plates were covered and maintained at room temperature for many weeks until a complete fungal growth was noticed. Ascomata and mycelia were microscopically observed. Cultures were performed by seeding with a sterile needle onto Malt Agar and Potato Carrot Agar, then incubated at 25 °C, until a fungal growth was noticed. Fungal colonies were characterized according to their macro and microscopical features. Fungal species were recognized following the keys provided by de Hoog et al. [28] and by Rebell and Taplin [13] for dermatophytes, and by Carmichael [29] for *Chrysosporium* and related genera.

Water samples were processed to search for *Prototheca* spp. and potentially pathogenic yeasts. They were seeded onto Prototheca Isolation Medium (PIM) and Sabouraud dextrose agar, added with gentamicin and biphenyl (1‰ each), in duplicate, then incubated at 25 °C and 37 °C. Cultures were maintained in an incubator for 30 days, then discarded if negative. Mycotic growth was macro-microscopically evaluated, and subcultures were achieved to yield pure fungal growth for a taxonomic characterization. Yeasts were also tested for carbohydrate assimilation by ID (bioMérieux, France). Moreover, auxanographic results were confirmed by PCR and sequencing. Total DNA was extracted from 20 mg cultured yeasts using the Quick-DNA Fungal/Bacterial Kit (Zymo Research, Irvine, CA, USA), following the manufacturer’s instructions. A fragment of the ITS gene region was amplified by PCR using the primers MalaITS1 (TCCGTAGGTGAACCTGCGG)-MalaITS4 (TCCTCCGCTTATTGATATGC) [30]. Amplified PCR products were sent for Sanger sequencing to an external company. Sequences were compared against those deposited in GenBank by using the National Center for Biotechnology Information (NCBI) Basic Local Alignment Search Tool (BLAST).

## 3. Results

Eighty-three fecal specimens were found in 23/26 (88.5%) parks.

### 3.1. Bacteriological Examinations

All fecal samples were negative for *Salmonella* spp. and *Campylobacter* spp. Seven samples (8.4%) were positive for *Yersinia* spp.: seven isolates (1 *Y. enterocolitica* biotype 1, 2 *Y. enterocolitica* biotype 2, 4 *Y. frederiksenii*) were cultured. Four (4.8%) strains of *Listeria innocua* were isolated from four fecal samples.

### 3.2. Parasitological Examinations

Only three stool specimens (3.6%) scored positive for helminth. In detail, *T. canis* eggs were identified in two samples (2.4%) and *Ancylostoma/Uncinaria* in the other (1.2%).

### 3.3. Mycological Examinations

Keratinophilic fungi were isolated from 16/26 (61.5%) areas. *Microsporum gypseum* (*A. incurvatum*) was the most recovered fungal species, being present in 11/16 soil specimens, alone (six specimens) and in association with *Microsporum cookei* (*Arthroderma cajetani*) (two specimens), with *Trichophyton terrestre* (*Arthroderma quadrifidum*), with *Chrysosporium indicum* (*Aphanoascus terreus*) and *Microsporum canis* (one specimen each). Two *Trichophyton ajelloi* (*Arthroderma uncinatum*), one *T. terrestre* and one *Chrysosporium keratinophilum* (*Aphanoascus fulvescens*) were also isolated. Feathers were the most frequently colonized baits, followed by horsehair. However, interestingly, *M. gypseum* isolated from two areas showed a striking growth on human hair in comparison with animal hair baits.

*Prototheca* spp. was never cultured from water specimens, while *Trichosporon* sp. was isolated from two samples, alone (Villa Favard) and in association with *Geotrichum* candidum (Anconella).

Microbiological, parasitological and mycological findings are summarized in Table 2 and Table 3.

## 4. Discussion

The present survey evaluated the occurrence of different bacterial, fungal, and helminthic pathogens in canine feces and environmental (soil and water) samples, with the aim to verify the contamination degree of public off-leash parks present in an important city located in central Italy, with a high density of human population and many pet dogs. The presence of dog stools in almost 88.5% of examined parks agrees with previous similar studies, where an occurrence of 98.6% and 86% were recorded in Naples and Milan, respectively [31,32].

*Yersinia* isolates were cultured from 8.4% of the analyzed fecal samples, and among them, 42.9% were *Y. enterocolitica*. A previous study carried out on several canine fecal samples collected from different European countries found a 4.6% prevalence of *Y. enterocolitica*, and in 63.6% of these cases, *Y. enterocolitica* was isolated as the only enteropathogenic bacterium. In the remaining 36.4% of the cases, other enteropathogenic bacteria were simultaneously detected, mainly *Salmonella* sp. and *Campylobacter* sp. [33].

Since 1980, investigations carried out in Europe found prevalence values ≤5% for *Yersinia* sp. and up to 30% for *Y. enterocolitica* in canine feces [23,34,35,36,37]. Data about the occurrence of *Yersinia* spp. in dogs in Italy are very limited. Fantasia and collaborators [37] isolated 17 strains of *Y. enterocolitica* biotype 4 from 63 puppies of an Italian kennel. During another investigation, one and four *Y. enterocolitica* strains were cultured from 212 dog feces and 240 soil specimens, respectively, collected from public areas in Southern Italy [35]. More recently, Cinquepalmi et al. [38] did not isolate *Yersinia* strains from 418 tested canine fecal samples.

*Yersinia*-infected dogs may develop enteric diseases of various severities, but asymptomatic carrier status seems to be predominant, and for this reason, *Y. enterocolitica* is considered a commensal in the intestine of these animals [33]. Asymptomatic infected dogs represent a serious threat in the epidemiology of yersiniosis because they are not recognized as infected, so they are not treated and can contaminate the environment with their feces, becoming a source of infection for owners and other animals.

In fact, companion animals have been considered as a potential source for human *Yersinia* infections because of their close contact [33,34,39]. Infected dogs may excrete yersiniae in their feces for more than 3 weeks at high cell counts [40]. Dogs may become infected by *Y. enterocolitica* through feeding and/or social interactions with other dogs; furthermore, it is assumed that raw pork is one of the most important sources of infection for them [41].

Our study did not find *L. monocytogenes* in the tested samples but detected *L. innocua* in four specimens with a 4.8% positivity. Studies on the presence of *Listeria* sp. in canine feces are scant. However, prevalence ranging from 0.92% to 11.53% have been detected in different countries [10,42,43,44,45]. In a recent investigation by Abay et al. [10], rectal swabs collected from 80 stray dogs admitted to the municipal kennel of Kayseri, Turkey, were examined: *Listeria* spp. were isolated from five (6.25%) samples, and one strain was identified as *L. monocytogenes*, whereas the remaining four were *L. innocua. L. innocua* is considered non-pathogenic for humans and animals, even though it has been occasionally isolated from them [46,47,48].

Our results, in agreement with those reported in the literature, confirmed that dogs can harbor *Listeria* sp. in the gastrointestinal tract and excrete the bacteria with their feces. Thus, pet dogs might be a source of infection for humans mainly through the consumption of food or water contaminated with canine feces and/or through direct contact with dogs.

*Salmonella* spp. was not found in the examined samples. These results are in accordance with previous data from the literature. In southern Italy, Cinquepalmi et al. [38] did not isolate salmonellae in the feces of 418 dogs, and Tarsitano et al. [49] did not find *Salmonella* spp. in 152 canine fecal samples collected in different public spaces. Moreover, other surveys carried to verify the occurrence of *Salmonella* spp. in healthy dogs found low prevalences [50,51,52].

No dogs were found positive for *Campylobacter* sp. in our investigation. These results are in accordance with Cinquepalmi et al. [38], who did not isolate *Campylobacter* strains from the canine fecal samples examined, but they are very different from those reported in other studies where different prevalences were detected [51]. Results are strongly related to several factors, such as canine populations and features of the collected samples. *Campylobacter* is difficult to culture from non-fresh fecal specimens; thus, our negative results could not reflect the real situation. In this study, in fact, feces were collected from the park areas and not directly from dogs. Even though fecal mass with fresh aspects was chosen, and the parks were regularly cleaned, samples may have not been recently excreted by the animals. The main issues with the fecal cultures are related to the microaerophilic character of *Campylobacter*; the time between specimen collection and culture setup thus becomes an uncontrolled variable in the ability to detect viable Campylobacter spp. by culture. Moreover, the bacteria, when stressed, may remain viable even though non-culturable [53].

*T. canis* eggs we found in 2.4% of the fecal samples. This positivity rate is very similar to the prevalence of 3.9% found in Portugal [54], while it is higher than data from Sassari (0.9%), Padua and Rome (1.9%), Milan (1.7%) and Naples (0.7%) [31,32,55,56]. Hookworm eggs were found in only one specimen, and the positivity rate was lower than the prevalence reported by Tamponi et al. [55] and Rinaldi et al. [31] (11.1% and 2.4%). Furthermore, *T. vulpis* eggs were never found. The main source of these parasites is represented by puppies and their lactating mothers [11], but these animals usually do not attend dog parks before their vaccination plan has been accomplished [57]. In general, the number of positive samples for a helminthic infection found in the present study appears rather low, although processed specimens cannot be considered too old to affect the testing sensitivity. 

Data from this survey could underestimate the occurrence of helminths. To exclude the presence of parasites, in fact, three fecal samples on three consecutive days should be processed [26], and it was not possible to trace the dogs.

The isolation of teleomorphic and anamorphic status of several geophilic keratinophilic fungi is not a surprising finding and agrees with previous similar studies from Italy [27,58], confirming the occurrence of these fungal species in areas where keratin debris are shed by animals. The only true geophilic dermatophyte responsible for tinea was *M. gypseum*, frequently involved in *tinea capitis*, *tinea corporis*, kerion and several other dermatologic diseases in human patients [59,60]. It has been largely reported from animals living in cities, too [61,62,63]. Furthermore, the apparent preference of some isolates for human hair would suggest more feasibility to people infection. Other fungal species such as *M. cookei* and *Chrysosporium* spp. have rarely been involved in superficial canine infections [64,65], while *T. ajelloi* occurs in an environment frequented by dogs and cats [66].

The growth of *M. canis* colonies on horsehair could indicate the presence of infected animals shedding arthrospores into the environment, being this dermatophyte a zoophilic species, not able to grow and mate off-host. As these fungal elements are proven to remain viable up to more than 30 months in the environment [67], the attendance of off-leash dog parks would pose a threat for ringworm in both animals and human beings. *M. canis* is responsible for *tinea corporis* [68] and for *tinea capitis* in children [69] and is the most frequent fungal species isolated from pets [61,62,63]. 

Water samples scored negative for *Prototheca* sp., and only *Trichosporon* sp and *Geotrichum candidum* were recovered from two puddles. The water specimens selected around the fountain basins scored negative for fungal growth, probably because of the good water renewal due to the daily use of fountains. Furthermore, these water specimens consisted of chlorinated water, which may be is not a good pabulum for these organisms. *Trichosporon* sp. and *G. candidum* are environmental opportunistic fungi, only seldom responsible for disease both in immunocompromised people [70]. Yeast belonging to genus *Trichosporon* was recently isolated from the oral mucosa of stray dogs [71]; furthermore, *Trichosporon jirovecii*, together with *Rhodotorula mucilaginosa*, was recently isolated from a dog with bronchotracheitis [72]. *G. candidum* was responsible for canine oral ulcers [73], cutaneous [74] and disseminate mycosis [75].

## 5. Conclusions

Off-leash dog parks are very important public areas where dogs living in urban districts may have a regular motorial activity that is necessary for a normal psycho-physical status. However, considering the large number of dogs that, with their owners, attend these confined public spaces, off-leash dog areas could favor the transmission of parasitic and infectious agents, including the zoonotic ones.

For this reason, it is important that owners are informed about the need and obligation to collect and properly dispose of the feces of their dogs. Veterinarians play a fundamental role in the education of dog owners to correctly follow hygiene and conduct standards. Moreover, veterinarians should inform owners of the importance of preventive medicine based on deworming treatments and regular fecal analyses.

Even though the present is not a longitudinal study, the results obtained would suggest that pet dogs attending examined parks are not common spreaders of intestinal pathogens, may be consequently to routine controls by veterinarians and prophylaxis against parasites.

However, in a One-Health perspective, periodical examinations to detect the main bacteriological, parasitological and mycological pathogens in different samples collected in off-leash dog parks might contribute to monitoring the contamination degree of these areas and the risk of transmission of pathogens to animals and humans.

## Figures and Tables

**Table 1:** Main characters of the off-leash parks located in different urban districts where samples were collected.

Urban District	Off-Leash Park	M^2^	Exposure	Trees	Soil
Q1	Porta Romana	553	Shady	maples	clay
Q2	Mezzetta	4141	Sunny	pines, limes	grass
Q2	Malta	579	Shady	pines, tuje	clay
Q2	Villa Favard	1300	Shady	holm oaks	grass
Q2	Tempio	680	Shady	holm oaks	clay
Q2	Moro	659	Sunny	cedars	grass
Q2	Venosta	522	Sunny	prunus	grass
Q2	Rocca Tedalda	100	Shady	limes	grass
Q2	Campo di Marte	15,043	Sunny	bangolari, limes, holm oaks	grass
Q2	Fanti	450	Shady	plane trees	grass
Q2	D’Ancona	124	Shady	maples	grass
Q3	Anconella	2361	Sunny	poplars, maples	grass
Q3	Villamagna	4141	Sunny	limes, Judas’s tree	grass
Q3	Gran Bretagna	873	Shady	pines	grass
Q3	Ponte a Ema	1100	Sunny	limes, cypresses, olive trees	grass
Q3	Pozzolatico	3235	Shady	poplars	grass
Q3	Isonzo	715	Sunny	mulberries, limes, olive trees	grass
Q3	Galluzzo	1345	Shady	pines	grass
Q4	Aleardi	1059	Sunny	limes, magnolia, holm oaks	grass
Q4	Strozzi	1500	Sunny	olive trees, birches, oaks, pines,	grass
Q4	Pisano	1659	Shady	plane trees	grass
Q4	S. Lorenzo a Greve	355	Sunny	maples	grass
Q5	Parnaso	2078	Sunny	holm oaks, birches, limes	grass
Q5	Zucchi	250	Sunny	no	grass
Q5	Circondaria	872	Shady	plane trees	grass
Q5	Pisacane	2919	Sunny	holm oaks, cypresses, olive trees	grass

**Table 2:** The results of microbiological and parasitological analysis on fecal samples collected in the examined areas.

Urban District	Off-Leash Park	Fecal Samples	Microbial Strains (Positive Samples)	Parasite Eggs (Positive Samples)
Q1	Porta Romana	1		
Q2	Mezzetta	9	*Listeria innocua* (3)	
Q2	Malta	2		
Q2	Villa Favard	5		
Q2	Tempio	1	*Yersinia frederiksenii* (1)	
Q2	Moro	4		
Q2	Venosta	6	*Yersinia enterocolitica* BT2 (1)	*Toxocara canis* (1),
Q2	Rocca Tedalda	5		*Ancylostoma caninum/Uncinaria stenocephala*(1)
Q2	Campo di Marte	1		
Q2	Fanti	3		
Q2	D’Ancona	1		*Toxocara canis* (1)
Q3	Anconella	7	*Listeria innocua* (1)	
Q3	Villamagna	11		
Q3	Gran Bretagna	2	*Yersinia frederiksenii* (1)	
Q3	Ponte a Ema	no		
Q3	Pozzolatico	1		
Q3	Isonzo	3		
Q3	Galluzzo	3		
Q4	Aleardi	5		
Q4	Strozzi	2	*Yersinia enterocolitica* BT2 (1)	
Q4	Pisano	no		
Q4	S. Lorenzo a Greve	3		
Q5	Parnaso	1	*Yersinia enterocolitica* BT1 (1)	
Q5	Zucchi	no		
Q5	Circondaria	3		
Q5	Pisacane	4	*Yersinia frederiksenii* (2)	

**Table 3:** The results of mycological analysis performed by hair bait technique on soil samples from selected areas.

Urban District	Off-leash Park	N. Soil Samples	Fungal Isolates	Hair	Horsehair	Feathers
Q2	Mezzetta	1	*Trichophyton terrestre/ Arthroderma quadrifidum*	0/1	1/1	1/1
Q2	Malta	1	*Microsporum gypseum/Arthroderma incurvatum*	1/1	1/1	1/1
Q2	Tempio	1	*Microsporum gypseum/Arthroderma incurvatum*	1/1	1/1	1/1
Q2	Moro	2	*Trichophyton terrestre/*(*Arthroderma quadrifidum*	1/2	1/2	2/2
Q2	Venosta	1	*Microsporum gypseum/Arthroderma incurvatum* *Microsporum cookie/Arthroderma cajetani*	0/1 0/1	1/1 1/1	0/1 1/1
Q2	Rocca Tedalda	1	*Trichophyton terrestre/ Arthroderma quadrifidum* *Microsporum gypseum/Arthroderma incurvatum*	0/1 1/1	1/1 1/1	0/1 1/1
Q3	Anconella	1	*Microsporum gypseum/Arthroderma incurvatum*	0/1	1/1	1/1
Q3	Villamagna	2	*Trichophyton ajelloi /Arthroderma uncinatum*	0/2	0/2	1/2
Q3	Ponte a Ema	2	*Trichophyton ajelloi /Arthroderma uncinatum*	1/2	2/2	2/2
Q3	Pozzolatico	1	*Chrysosporium keratinophilum/Aphanoascus fulvescens*	0/1	0/1	1/1
Q3	Isonzo	1	*Microsporum gypseum/Arthroderma incurvatum*	0/1	1/1	1/1
Q4	Aleardi	2	*Microsporum gypseum/Arthroderma incurvatum Microsporum cookie/Arthroderma cajetani*	0/2 0/2	2/2 2/2	1/2 1/2
Q4	Strozzi	3 *	*Microsporum gypseum/Arthroderma incurvatum*	0/3	3/3	2/3
Q4	Pisano	2	*Chrysosporium indicum/Aphanoascus terreus* *Microsporum gypseum/Arthroderma incurvatum*	0/2 1/2	0/2 2/2	2/2 2/2
Q5	Parnaso	3	*Microsporum gypseum/Arthroderma incurvatum*	2/3	3/3	2/3
Q5	Circondaria	4	*Microsporum gypseum/Arthroderma incurvatum*	4/4	3/4	1/4
total		43		8	14	16

* Legend–*Microsporum canis* was cultured from 1 soil specimen out of 3 on horsehair.

## Data Availability

Data are contained within the article.
